# Alzheimer’s disease diagnosis based on gene selection and cross-tissue validation

**DOI:** 10.3389/fgene.2026.1890236

**Published:** 2026-09-28

**Authors:** Xiaolan Zhang, Xiaotong Tang, Qian Bo, Yu Ma, Fang Shi, Hongpeng Hou

**Affiliations:** The Sixth Division Hospital of the Xinjiang Production and Construction Corps, Wujiaqu, China

**Keywords:** Alzheimer’s disease, biomarker, early diagnosis, gene selection, single-nucleus RNA sequencing

## Abstract

**Background:**

Early and accurate diagnosis is critical for intervening in the progression of Alzheimer’s disease (AD). However, traditional clinical diagnostic techniques, such as invasive cerebrospinal fluid punctures and expensive neuroimaging, struggle to meet the demands of large-scale early screening.

**Methods:**

This study proposes a novel transcriptomic-based diagnostic framework named DeepFS, which features an automated gene selection module to filter out redundant noise and identify high-value core gene targets for diagnosis.

**Results:**

DeepFS achieved high-precision identification of AD, yielding an ac-curacy of 89.45% and an AUC value of 0.9647 on brain tissue transcriptomic data. To further explore the methodological generalizability of the framework across different tissue types, we extended DeepFS to a peripheral blood transcriptomic dataset, where it consistently achieved reliable diagnostic classification performance. Furthermore, regarding the brain tissue transcriptomic data, the high-weight genes driven by the gene selection module were integrated with AD brain single-nucleus RNA sequencing data for quantitative analysis at single-cell resolution, further validating the biological efficacy of the module.

**Conclusion:**

DeepFS not only exhibits competitive performance in AD diagnostic classification, but can also serve as a valuable prior reference for future AD biomarker research.

## 1 Introduction

Alzheimer’s disease (AD) is a neurodegenerative disease with insidious onset and progressive development, characterized by cognitive impairment and non-cognitive neuropsychiatric symptoms, including impaired memory, judgment, and attention, personality abnormalities, behavioral abnormalities, and reduced ability to perform daily living and social activities (Porsteinsson et al., 2021; Vaz and Silvestre, 2020).

AD is the most common cause of dementia, accounting for 60%–80% of all dementias (Tatulian, 2022). The prevalence of AD is 4%–7% among the elderly over 65 years of age, and is the fifth leading cause of death among the elderly (Vlachos et al., 2020). Currently there is no drug that can treat AD, the existing drugs can only delay the patient’s condition and try to ensure the patient’s quality of life, therefore, early diagnosis of AD, early treatment is quite important.

AD affects the central nervous system through the accumulation of neurofibrillary tau tangles and beta-amyloid plaques in neurons, and newer magnetic resonance imaging (MRI), positron emission tomography (PET), and cerebrospinal fluid analyses are now commonly used diagnostic tools (Ausó et al., 2020). Ji et al. combined a convolutional neural network with MRI to achieve early diagnosis of AD using image slices of gray and white matter from MRI as input for classification (Ji et al., 2019). Zhang et al. trained multimodal medical images using two independent convolutional neural networks to ensure output consistency, and combined the output with clinical neuropsychology to achieve a diagnosis of AD (Zhang et al., 2019). Amoroso et al. used MRI data to diagnose AD by first performing feature selection through random forests and then feeding the data into a deep neural network for classification (Amoroso et al., 2018). Huang et al. used a 3D convolutional neural network to integrate MRI and PET data and diagnose AD through a fully connected layer (Huang et al., 2019). Kokkinou et al. demonstrated the accuracy of beta-amyloid in distinguishing Alzheimer’s dementia from specific dementia types based on the fact that beta-amyloid is reduced in the cerebrospinal fluid of patients with dementia in AD relative to patients without dementia (Kokkinou et al., 2021).

Although the commonly used diagnostic tools mentioned above offer more possibilities for early diagnosis of AD, they suffer from several limitations in practical clinical application. First, they are costly and unsuitable for mass screening. Second, cerebrospinal fluid testing carries certain risks and discomfort, limiting patient acceptance (Ausó et al., 2020). Thus, while these techniques have great potential for scientific research and diagnostic accuracy, their invasiveness and high cost remain important challenges for the medical community and patients. In contrast, transcriptomics is non-invasive, potentially widely applicable, and relatively low-cost, and its application can alleviate these challenges in AD diagnosis to some extent (Iturria-Medina et al., 2022).

We developed DeepFS, a gene selection-based framework for AD diagnosis from transcriptomic data. DeepFS integrates a supervised gene selection module directly into the input layer of a deep neural network. Specifically, the module employs a learnable gating tensor with the same dimensionality as the input gene expression profile, acting as a soft feature mask. During training, the gating tensor is jointly optimized with the downstream classifier *via* backpropagation, allowing the contribution of each gene to be adaptively adjusted according to the classification objective. In contrast to embedded feature selection approaches such as LASSO, Elastic Net, and Random Forest feature importance, DeepFS does not depend on predefined linear assumptions or tree-based splitting criteria. Instead, gene selection is seamlessly integrated into the deep learning framework and optimized jointly with the classifier. Consequently, feature selection is directly driven by the end-task objective rather than an independent feature evaluation criterion. Moreover, by exploiting the nonlinear modeling capacity of deep neural networks, DeepFS is capable of capturing complex higher-order gene interactions, thereby achieving end-to-end joint optimization of gene selection and AD classification.

Furthermore, sparsity is imposed explicitly on the gating tensor rather than on the entire network parameters, enabling irrelevant genes to be suppressed while preserving the nonlinear modeling capacity of the downstream classifier. By decoupling gene selection from nonlinear decision modeling through a dedicated gating layer, DeepFS achieves improved interpretability and more stable feature selection compared with conventional regularized MLP-based approaches.

In this study, we first extracted the differential expressed genes between AD and controls by differential expression analysis, then input them into the gene selection module to choose the key genes, and realized the diagnosis of AD by multilayer perceptron at the end. To explore the methodological generalizability of the framework across different tissue types, we further independently retrained and validated DeepFS on a peripheral blood transcriptomic dataset. Furthermore, regarding the brain tissue transcriptomic data, the high-weight genes driven by the gene selection module were integrated with AD brain single-nucleus RNA sequencing (snRNA-seq) data for quantitative analysis at single-cell resolution, further validating the biological efficacy of the module. The workflow is shown in Figure 1.

**FIGURE 1:**
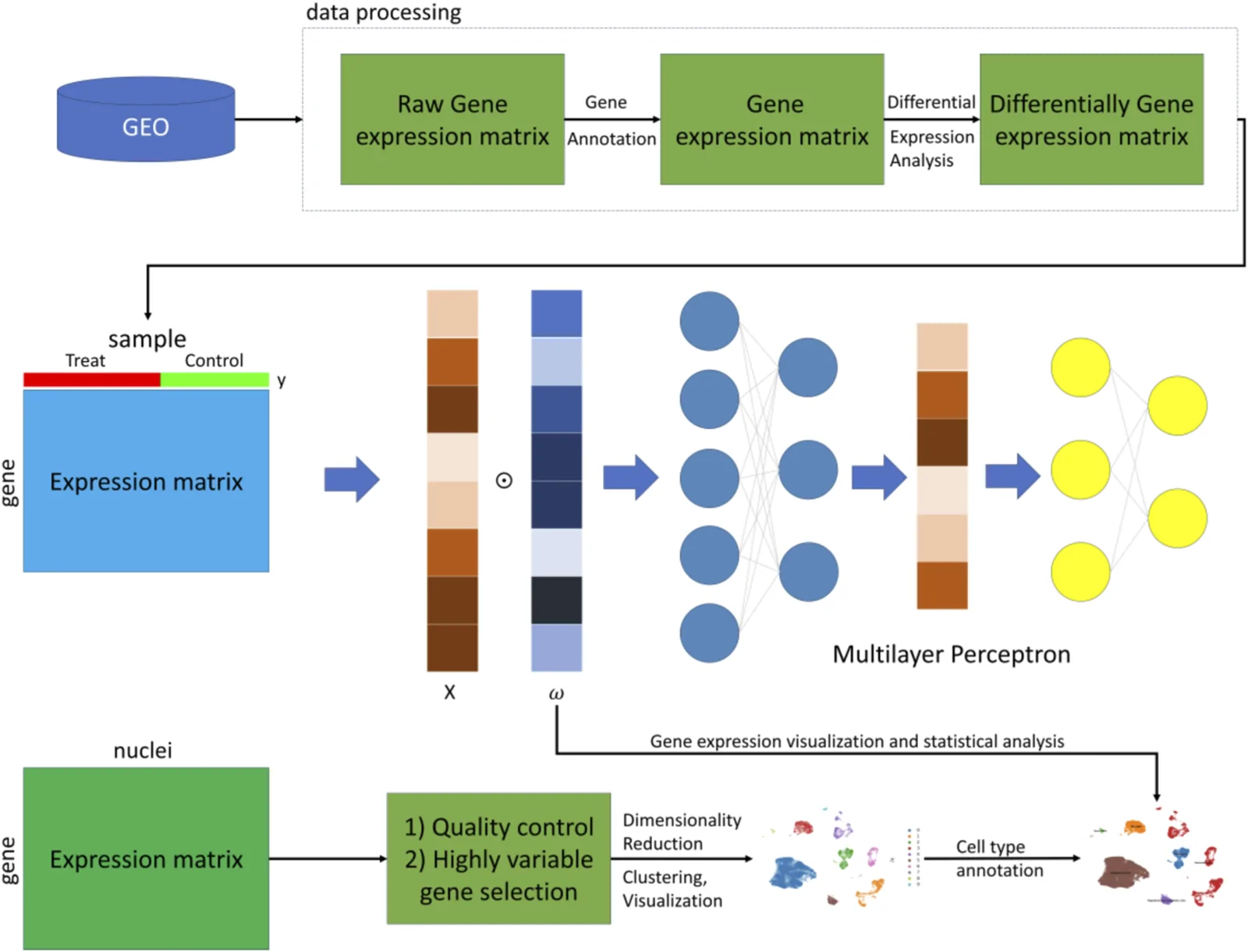
Workflow Diagram. For bulk transcriptomics data, probes were first annotated and mapped to gene symbols based on the platform files. Subsequently, the dataset was partitioned using 5-fold cross-validation strategy. Differential expression analysis was then performed, and the resulting feature matrices were fed into the DeepFS framework for pathological classification. For the snRNA-seq data, the analytical pipeline commenced with quality control, followed by the selection of highly variable genes, dimensionality reduction, clustering, and cell type annotation. Finally, cell-type-specific differential expression analysis was conducted to facilitate cross-modality biological validation.

## 2 Materials and methods

### 2.1 Data

The human brain tissue transcriptomic data were obtained from the GEO database (https://www.ncbi.nlm.nih.gov/geo/query/acc.cgi?acc=GSE5281), comprising 161 samples consisting of 87 AD samples and 74 control samples. After gene annotation of the original expression matrix and elimination of duplicate genes by the platform gene annotation file, a final gene expression matrix with 23,348 genes and 161 samples was obtained. Given the characteristic of the dataset containing samples from different brain regions belonging to the same donor, we conducted subject-level cross-validation during the cross-validation phase. For 5-fold cross-validation, samples were grouped according to subject ID to prevent data leakage. All samples from the same subject, including those obtained from different brain regions, were assigned to the same fold and were therefore present exclusively in either the training or test set in each fold. The human peripheral blood transcriptome data were obtained from the GEO database (https://www.ncbi.nlm.nih.gov/geo/query/acc.cgi?acc=GSE63060), comprising 249 samples consisting of 145 AD samples and 104 control samples. After gene annotation of the original expression matrix and elimination of duplicate genes by the platform gene annotation file, a final gene expression matrix with 21,696 genes and 249 samples was obtained.

In both datasets, when multiple probes mapped to the same gene, their expression values were averaged using the avereps function in the limma package to generate a unique gene-level expression matrix. Subsequently, log2 transformation h[log2(x+1)] and between-array normalization were performed using the limma package.

The human brain tissue snRNA-seq data were obtained from the GEO database (https://www.ncbi.nlm.nih.gov/geo/query/acc.cgi?acc=GSE174367), comprising 18 samples consisting of 11 AD samples and 7 control samples. During the initial data preprocessing, nuclei lacking corresponding clinical diagnostic metadata were excluded. Subsequently, rigorous quality control was applied to the single-nucleus dataset: at the nucleus level, only high-quality nuclei with the number of detected genes ranging from 200 to 7,500 and total UMI counts exceeding 1,000 were retained, while those with a mitochondrial gene fraction of over 20% were filtered out; at the gene level, low-frequency genes expressed in fewer than 3 nuclei were eliminated. Ultimately, a dataset comprising 58,363 nuclei and 35,429 genes was retained for downstream analysis.

### 2.2 Differential expression analysis

Differential expression analysis (DEA) is commonly used for transcriptomics data and is mainly used to compare gene expression levels under different conditions (e.g., disease vs. health status, treatment vs. control, *etc.*) and to identify genes that show significant differences in different conditions (Anders and Huber, 2010). In this study, we used the limma package (Ritchie et al., 2015) in R to perform DEA between AD samples and control samples, set |logFC| > 1.0 and adj.P.Val <0.05 to screen the differentially expressed genes and generate the differential expression matrices, and in order to visualize the expression of these differentially expressed genes, we further used the R package pheatmap to plot the heatmap of gene expression, in addition, volcano plots of the differential genes were also drawn by ggplot2 to visualize the expression folds of the genes with the significance levels, thus intuitively presenting the distribution of genes that were significantly upregulated or downregulated between the AD samples and the control group.

### 2.3 DeepFS

In this study, we propose DeepFS, a deep learning framework based on gene selection for AD diagnosis, which consists of a gene selection module and a multilayer perceptron (MLP). The gene selection module is initialized as a random tensor and normalized, and then encapsulated as a learnable weight parameter. The presence of the gene selection module enables DeepFS to perform gene selection during the training process to identify informative genes, thereby allowing for the learning of an appropriate gene space and ultimately facilitating the precise diagnosis of AD. The input to the MLP is the gene expression data of the sample after performing gene selection, which consists of one input layer, two hidden layers and one output layer to perform the diagnosis of AD.

### Gene selection module

To adaptively identify key biological markers, we introduce a selection weight $\omega \in R^{N}$ . The selection weight vector ω is initialized following a scaled uniform distribution to ensure numerical stability during the initial training phase. Specifically, each element of ω is sampled from a uniform distribution $U\left(0,1\right)$ and subsequently normalized by the square root of the input dimension N as shown in Equation 1. This initialization scheme, inspired by the Xavier method, prevents the internal activations from saturating and facilitates a more stable gradient flow through the selection layer, especially when dealing with high-dimensional transcriptomics data.

$$\omega ∼U\left(0,\frac{1}{\sqrt{N}}\right)$$ (1)

Given the input feature matrix $X\in R^{M\times N}$ , the gated output $X_{out}$ is formulated as Equation 2:

$$X_{out}=X⊙\omega$$ (2)

where ⊙ denotes the element-wise multiplication (Hadamard product). The selection weights are learned during the training process to capture the relative importance of each feature.

### Multilayer perceptron

After the gene selection, the filtered data $X_{out}$ is fed into a multilayer perceptron to learn high-level abstract representations. Specifically, the MLP comprises two hidden layers with h units each (where h = hidden_size). Unlike the standard ReLU, the ELU activation is utilized to provide smoother gradients and allow for negative outputs, which helps the model push the mean activations closer to zero and speed up convergence. The propagation process is shown in Equation 3.

$$H=\sigma \left(W_{2}\sigma \left(W_{1}X_{out}+b_{1}\right)+b_{2}\right)$$ (3)

where σ denotes the ELU activation, and H represents the extracted hidden representations.

### Diagnostic classification module

To obtain the final diagnostic results, the high-level latent representations extracted by the MLP are projected onto a 2-dimensional decision space through a linear output layer. Subsequently, a Softmax activation function is applied to the resulting logits to produce a normalized probability distribution over the target classes. The Softmax operation ensures that the sum of the outputs equals one, allowing the model to provide a probabilistic confidence score for each diagnosis. This final transformation is defined as Equations 4 and 5:

$$z=W_{out}H+b_{out}$$ (4)

$${\hat{y}}_{i}=\frac{e^{z_{i}}}{\sum_{j=1}^{2}e^{z_{j}}},i\in \left\{0,1\right\}$$ (5)

where ${\hat{y}}_{i}$ represents the predicted probability that the sample belongs to the *i*th category.

The objective function of DeepFS is as follows Equation 6:

$$L=cross\_entropy\left(\hat{y},y\right)+\gamma {\left\|\omega \right\|}_{1}$$ (6)

The cross-entropy loss function is used in the training process, where ŷ is the prediction score of DeepFS, y is the label corresponding to the sample, and in order to make the gene selection module have the function of feature selection, we perform L1 regularization, which encourages sparsity. ω is the weight parameter in the gene selection module, and γ are the corresponding regularization coefficients.

### ScmpImpute implementation

DeepFS is implemented using Python 3.9 and PyTorch 2.7.0 on an NVIDIA GeForce RTX 5060 GPU. The network is trained using the Adam optimizer with a learning rate of 0.0003, a hidden layer size of 64 neurons, and a total of 100 epochs. These hyperparameters were selected through grid search to identify the optimal configuration.

### 2.4 Enrichment analysis and PPI network construction

Gene ontologies are used to describe the functions of genes and gene products, which are divided into three main categories: biological processes, molecular functions, and cellular components (Consortium et al., 2023), whereas KEGG is a database that contains human pathways and related biological processes (Kanehisa et al., 2017). GO enrichment analysis can explain the function of a specific gene, and KEGG enrichment analysis can identify the relevant pathways involved in a specific gene, thus providing a deeper biological context for the results. In this study, we used differentially expressed genes for enrichment analysis, firstly, we converted gene symbols to Entrez gene IDs and performed enrichment analysis by R package clusterProfiler, and visualized the enrichment by bar chart and bubble chart to show the enrichment. Then we performed PPI network construction of differential genes by the online tool STRING (https://string-db.org/) to further understand the functions and interactions of these genes in cells.

### 2.5 Processing of single-nucleus RNA sequencing data

Highly Variable Gene (HVG) Selection. Following the quality control of the snRNA-seq dataset, we performed HVG selection to capture 3,000 highly variable genes. Crucially, all mitochondrial, ribosomal, and hemoglobin genes were thoroughly excluded from this feature space to prevent ambient background artifacts from confounding the downstream analyses (Liu and Li, 2023; Wang et al., 2022a).

Dimensionality Reduction, Clustering, and Visualization. Principal Component Analysis was first employed on the snRNA-seq dataset for dimensionality reduction. Subsequently, the Euclidean distances among nuclei were calculated based on the top 30 principal components to construct a neighborhood graph topological structure in the low-dimensional space. On this basis, the Leiden algorithm was applied for graph clustering to identify distinct cell lineages (Traag et al., 2019), and the Uniform Manifold Approximation and Projection (UMAP) algorithm was ultimately utilized for visualization.

Cell Type Annotation. First, DEA was performed to identify marker genes for distinct clusters. We strictly retained significantly differentially expressed genes that met the following criteria: an average log2 fold-change ( $\log_{2}FC$ ) > 0.6, a Benjamini-Hochberg adjusted P-value ( $P_{adj}$ ) < 0.05, and an expression frequency within the target cluster (pct_nz_group) > 0.25. Concurrently, mitochondrial and ribosomal genes were excluded. Ultimately, these marker genes from each cluster were cross-referenced with authoritative literature and canonical cell marker databases to achieve cell type annotation at single-cell resolution.

Quantitative Statistical Analysis. Utilizing the snRNA-seq dataset, cell-type-specific DEA was systematically executed between the AD and control groups within each distinct cell lineage. Benjamini–Hochberg procedure was used to correct for multiple testing, and $P_{adj}$ < 0.05 were used to assess statistical significance. This quantitative evaluation aimed to cross-examine whether the core genes, which were prioritized with high weights by the gene selection module in the bulk brain transcriptomic data, still exhibited statistically significant variations at single-cell resolution.

## 3 Results

### 3.1 Evaluation metrics

In this study, a 5-fold cross-validation was taken to evaluate the performance of DeepFS, and the evaluation metrics used include: accuracy (Acc), sensitivity (Sen), specificity (Spec), precision (Prec), Mathews correlation coefficient (MCC), F1 index, and Area Under the Receiver Operating Characteristic curve (AUC), and their formulas are shown below as Equations 7–12, and the average results of the 5 experiments were taken as the final results of the model in this study.

$$Acc=\frac{TP+TN}{TP+TN+FP+FN}$$ (7)

$$Sen=\frac{TP}{TP+FN}$$ (8)

$$Spec=\frac{TN}{TN+FP}$$ (9)

$$Prec=\frac{TP}{TP+FP}$$ (10)

$$MCC=\frac{TP\times TN-FP\times FN}{\sqrt{\left(TP+FP\right)\left(TP+FN\right)\left(TN+FP\right)\left(TN+FN\right)}}$$ (11)

$$F1=\frac{2\times Prec\times Sen}{Prec+Sen}$$ (12)

where TP denotes the number of samples correctly identified as AD, TN denotes the number of samples correctly identified as controls, FP denotes the number of samples incorrectly identified as AD, and FN denotes the number of samples incorrectly identified as controls.

### 3.2 Identification of differentially expressed genes

During the 5-fold cross-validation pipeline, the differential expression analysis (DEA) was strictly and independently conducted within each individual training fold. By intersecting the results, we prioritized a robust consensus core of 662 significantly differentially expressed genes that consistently recurred across all five iterations, which exhibited significant differences in expression levels between the two groups of samples and provided critical molecular clues for further biological investigations. In order to visualize the distribution of these differentially expressed genes more intuitively, we drew heatmaps and volcano diagrams to show the gene expression patterns and significance, respectively. These graphs are shown in Figure 2, where the heatmap clearly demonstrates the genes with significant expression changes between AD samples and controls, while the volcano plot emphasizes the statistical significance of the differentially expressed genes and their fold changes in expression.

**FIGURE 2:**
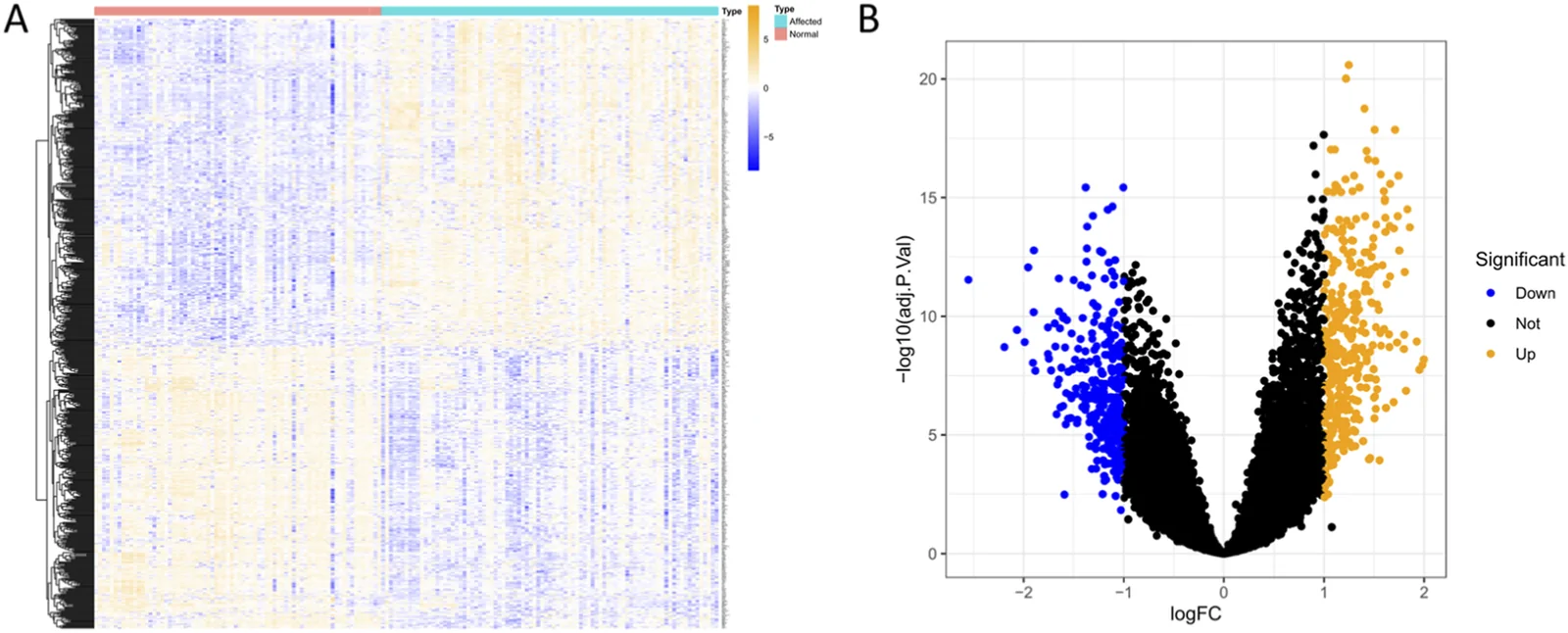
Visualization of DEA results. **(A)** Heatmap of differentially expressed genes. Rows represent genes, columns represent samples, and yellow indicates high expression and blue indicates low expression. **(B)** Differentially expressed gene volcano plot. Each point represents a gene, and the horizontal axis represents the multiplicity of differences, with the more off-center representing the greater multiplicity of differences. The vertical axis represents significance, the closer to the top means the more significant the difference is.

### 3.3 Model classification capabilities

In this study, the differential expression matrix was used as input to DeepFS to realize the classification of AD and control samples, and the experimental results of the five-fold cross-validation are shown in Table 1. As can be seen in Table 1, DeepFS obtained an Acc of 89.45% and an AUC value of 0.9647, with a Sen and Spec of 92.54% and 86.61%, and an Prec, MCC and F1 index of 88.76%, 78.91% and 90.40%, respectively. This demonstrates the competitive predictive ability of DeepFS and its potential in diagnosing AD using transcriptomic data. Meanwhile, this study visualized the cross-validated Receiver Operating Characteristic (ROC) curves, as shown in Figure 3.

**TABLE 1:** Diagnostic results of DeepFS.

Fold	Acc	Sen	Spec	Prec	MCC	F1	AUC
1	87.88%	88.89%	86.67%	88.89%	75.56%	88.89%	0.9185
2	90.62%	100.00%	81.25%	84.21%	82.72%	91.43%	0.9766
3	100.00%	100.00%	100.00%	100.00%	100.00%	100.00%	1.0000
4	81.25%	80.95%	81.82%	89.47%	60.70%	85.00%	0.9524
5	87.50%	92.86%	83.33%	81.25%	75.59%	86.67%	0.9762
Avg	89.45% ± 6%	92.54% ± 7%	86.61% ± 6%	88.76% ± 6%	78.91% ± 12%	90.40% ± 5%	0.9647 ± 0.02
95% CI	[80.98%, 97.92%]	[82.55%, 100%]	[76.96%, 96.27%]	[79.90%, 97.63%]	[60.83%, 96.74%]	[83.09%, 97.71%]	[0.9264, 1.0000]

**FIGURE 3:**
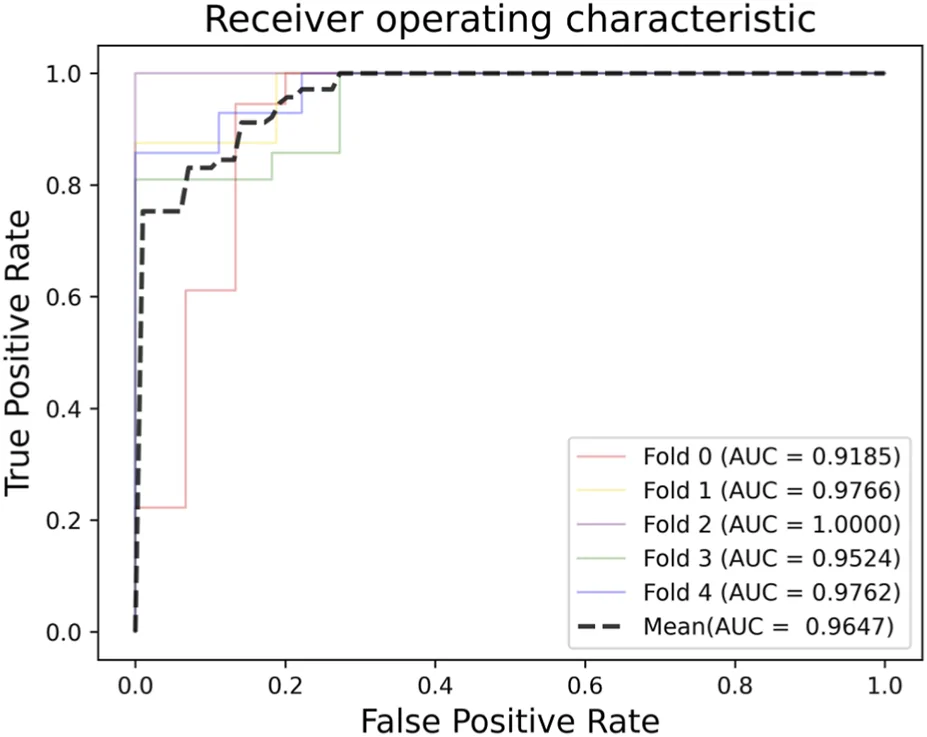
ROC curves of 5-fold cross-validation on brain transcriptomic data.

### 3.4 Model comparison

To evaluate the diagnostic performance of DeepFS for AD, we benchmarked it against standard algorithms, including Random Forest (RF), Support Vector Machine (SVM), Logistic Regression (LR), K-Nearest Neighbors (KNN), Extreme Gradient Boosting (XGBoost), Light Gradient Boosting Machine (LightGBM), Categorical Boosting (CatBoost) and DeepBrain (Wang Q. et al., 2022), a deep learning method for AD diagnosis using transcriptomic data. Furthermore, to demonstrate the effectiveness of the gene selection module, we replaced it in DeepFS with L1-regularized logistic regression (DeepFS_L1-LR) and Elastic Net (DeepFS_EN) and evaluated the resulting classification performance. All models were hyperparameter-tuned *via* grid search to achieve optimal results, and a 5-fold cross-validation scheme was employed to rigorously evaluate their diagnostic efficacy. The experimental results, detailed in Table 2, reveal that DeepFS consistently achieved optimal performance across the majority of metrics, yielding an Acc of 89.45% (±6%) and an AUC of 0.9647 (±0.02), outperforming the comparative methods. These results not only demonstrate the competitiveness of DeepFS for AD diagnosis but also further highlight the importance of the gene selection module. By jointly optimizing the gene selection module and the MLP within an end-to-end supervised framework, DeepFS can effectively extract latent discriminative features and capture representative patterns from complex, high-dimensional gene expression data.

**TABLE 2:** Results of model comparison.

Methods	Acc	Sen	Spec	Prec	MCC	F1	AUC
RF	80.11% ± 8%	70.87% ± 10%	91.99% ± 6%	91.09% ± 6%	63.30% ± 13%	79.25% ± 7%	0.8829 ± 0.06
SVM	68.32% ± 11%	99.05% ± 1%	35.01% ± 16%	64.04% ± 11%	45.09% ± 13%	77.04% ± 8%	0.9454 ± 0.03
LR	82.61% ± 5%	95.08% ± 7%	70.27% ± 9%	78.46% ± 8%	67.83% ± 8%	85.32% ± 4%	0.9432 ± 0.04
KNN	84.53% ± 6%	87.78% ± 8%	81.27% ± 6%	84.34% ± 6%	69.04% ± 12%	85.77% ± 5%	0.9521 ± 0.02
XGBoost	83.88% ± 7%	92.38% ± 5%	75.49% ± 11%	81.13% ± 10%	68.76% ± 12%	85.93% ± 6%	0.9485 ± 0.05
LightGBM	84.53% ± 8%	91.27% ± 6%	77.99% ± 12%	82.67% ± 11%	69.60% ± 16%	86.36% ± 7%	0.9235 ± 0.06
CatBoost	87.61% ± 7%	92.54% ± 7%	82.84% ± 9%	86.03% ± 8%	75.41% ± 14%	88.90% ± 6%	0.9564 ± 0.04
DeepBrain	87.58% ± 6%	91.29% ± 6%	83.37% ± 7%	86.64% ± 5%	74.64% ± 13%	88.81% ± 5%	0.9584 ± 0.03
DeepFS_L1-LR	85.17% ± 9%	86.03% ± 14%	85.81% ± 7%	86.78% ± 8%	71.25% ± 17%	85.88% ± 9%	0.9416 ± 0.04
DeepFS_EN	83.33% ± 11%	82.56% ± 17%	85.95% ± 9%	86.29% ± 11%	67.99% ± 21%	83.63% ± 12%	0.9384 ± 0.04
DeepFS	89.45% ± 6%	92.54% ± 7%	86.61% ± 6%	88.76% ± 6%	78.91% ± 12%	90.40% ± 5%	0.9647 ± 0.02

### 3.5 Enrichment analysis results and PPI network

In this study, GO enrichment analysis and KEGG enrichment analysis were performed for differentially expressed genes, and bubble charts and bar charts were drawn, as shown in Figures 4A,B. In the results of GO enrichment analysis, different expressed genes were significantly enriched in the reaction and homeostasis of copper, zinc and cadmium ions, while the correlation between AD and metal ions has been extensively studied, especially copper, zinc and iron ions play an important role in the pathogenesis of AD. Transition metals such as copper, zinc, and iron can participate in amyloid protein aggregation, oxidative stress, and synaptic dysfunction, and may contribute to AD pathogenesis by affecting neuronal homeostasis and protein metabolism. Therefore, the observed enrichment of metal ion homeostasis-related processes may reflect transcriptional alterations associated with redox imbalance, disrupted protein homeostasis, and neuronal dysfunction in AD brain tissue (Andrási et al., 1995; Hung et al., 2010; Hureau and Faller, 2009). These findings provide further support for the involvement of metal ion homeostasis in the molecular alterations associated with AD. In the results of KEGG enrichment analysis, the largest number of genes were enriched in the AD pathway, and also significantly enriched in synaptic vesicle circulation, GABAergic synapses and Huntington’s disease pathways, which are related to nerves, further confirming the heterogeneity and multi-factor characteristics of AD, and may provide new targets for future therapeutic strategies. The PPI network diagram drawn in this study is shown in Figure 4C. At the same time, we use the interaction frequency of genes in the PPI network to draw the ranking bar chart of key genes. The proteins encoded by these genes interact with many other proteins in the PPI network and have high linking degree, which may participate in multiple biological processes or pathways. Are potential biomarkers or therapeutic targets, as shown in Figure 4D.

**FIGURE 4:**
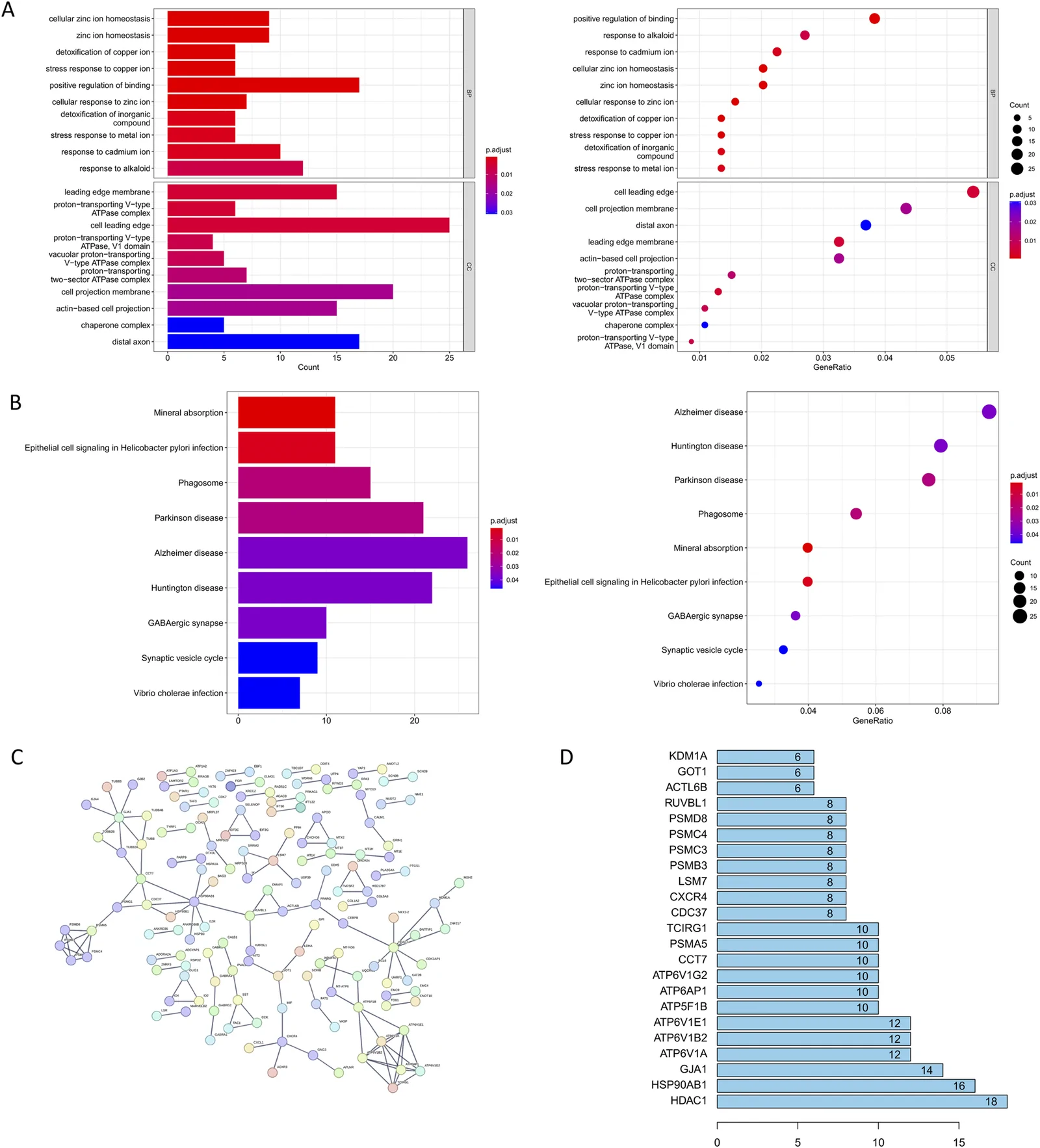
**(A)** GO enrichment analysis results. **(B)** KEGG enrichment analysis results. **(C)** PPI network mapped by differentially expressed genes. **(D)** Histogram of key gene rankings based on frequency of interactions in the PPI network.

### 3.6 Independent validation

Cross-Tissue Validation in Peripheral Blood. To further explore the methodological generalizability of the proposed framework across different tissue types, DeepFS was applied to the human peripheral blood transcriptomic data. The data preprocessing pipeline implemented for this cohort was identical to that used for the bulk brain transcriptomic data, and the detailed classification results are summarized in Table 3. Specifically, DeepFS achieved a classification accuracy of 78.74% and an AUC value of 0.8453. Given that AD is a primary central nervous system disorder with its pathological epicenter sequestered within the brain, brain-derived molecular signals are diluted in the circulatory system, typically resulting in a low signal-to-noise ratio in peripheral blood (Zetterberg, 2019). In this highly noisy peripheral context, DeepFS still demonstrated robust predictive capacity, securing a 0.84 AUC and an 85.81% Sen. This robust performance, particularly the low false-negative rate implied by the high sensitivity, suggests promising potential as a preliminary computational aid for exploratory screening and as a tentative reference for future early detection research. However, given the relatively moderate performance compared with that achieved using brain tissue and the limitations of the current study, further validation in larger, independent cohorts is required to determine its clinical applicability. We further conducted a literature review of the high-weight genes selected by DeepFS from the peripheral blood dataset. Among these genes, NDUFA1, RPS24, and RPL17 have previously been reported to be associated with AD (Zhang et al., 2025; Cao et al., 2024; Zhao et al., 2024), while RPL26 has also been reported as a promising potential blood biomarker for AD (Wang et al., 2024). These previous findings provide, to some extent, independent biological support for the high-weight genes selected by DeepFS from the peripheral blood dataset. The ROC curves derived from the cross-validation were visualized and are illustrated in Figure 5.

**TABLE 3:** Cross-tissue peripheral blood validation classification results of DeepFS.

Fold	Acc	Sen	Spec	Prec	MCC	F1	AUC
1	78.00%	86.21%	66.67%	78.12%	54.37%	81.97%	0.8555
2	68.00%	81.48%	52.17%	66.67%	35.41%	73.33%	0.7101
3	84.00%	79.41%	93.75%	96.43%	68.75%	87.10%	0.8887
4	78.00%	89.66%	61.90%	76.47%	54.55%	82.54%	0.8571
5	85.71%	92.31%	78.26%	82.76%	71.66%	87.27%	0.9147
Avg	78.74%	85.81%	70.55%	80.09%	56.95%	82.44%	0.8453

**FIGURE 5:**
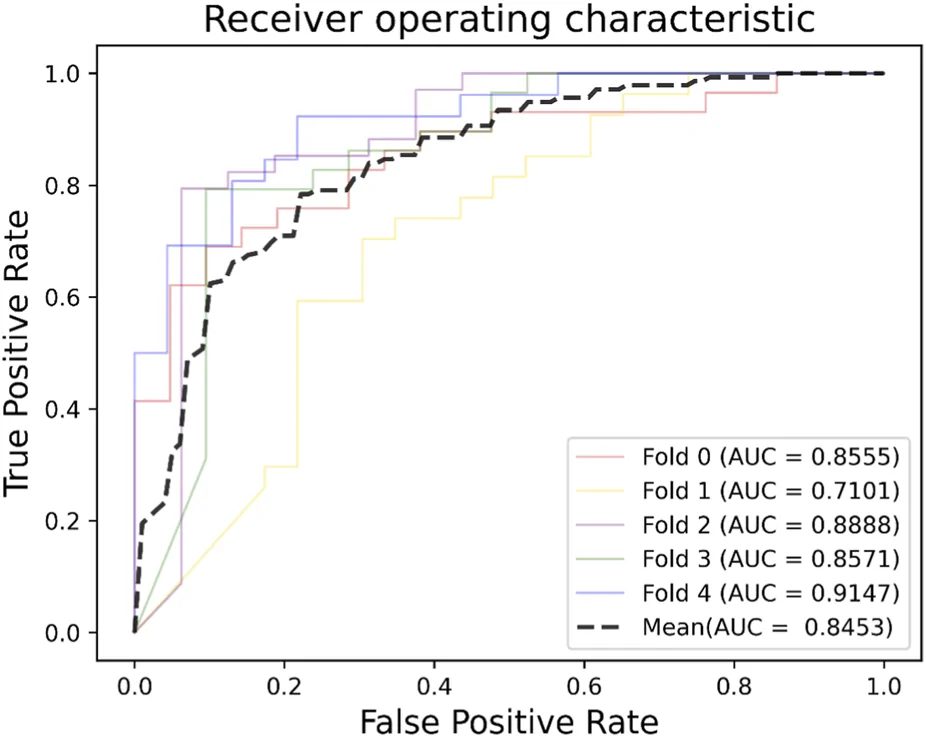
ROC curves of 5-fold cross-validation on peripheral blood transcriptomic data.

Single-Nucleus Sequencing Validation. To evaluate the biological validity of the gene selection module, this study further introduced an snRNA-seq dataset as an independent external validation set. This section aims to conduct a cross-platform, fine-scale, and multidimensional analysis of the core genes prioritized by the module at single-cell resolution. By investigating their cell-type-specific expression profiles within major brain cellular lineages, we aim to verify the effectiveness of the DeepFS gene selection module at a micro-scale level. Following the execution of quality control, dimensionality reduction, clustering, and cell type annotation, a total of 6 cell types were identified, including oligodendrocytes, microglia, oligodendrocyte progenitor cells, astrocytes, mural cells, and neurons. Statistics on the number of cell types are presented in Table 4, with the UMAP visualization illustrated in Figure 6B.

**TABLE 4:** Statistics on the number of cell types.

Cell type	AD samples	Control samples	AD nuclei	Control nuclei
Astrocytes	11	7	2,805	1,649
Microglia	11	7	2,585	1,300
Mural cells	11	7	275	147
Neuronal cells	11	7	7,127	2,767
Oligodendrocyte progenitor cells	11	7	1,680	1,008
Oligodendrocytes	11	7	21,846	14,835

**FIGURE 6:**
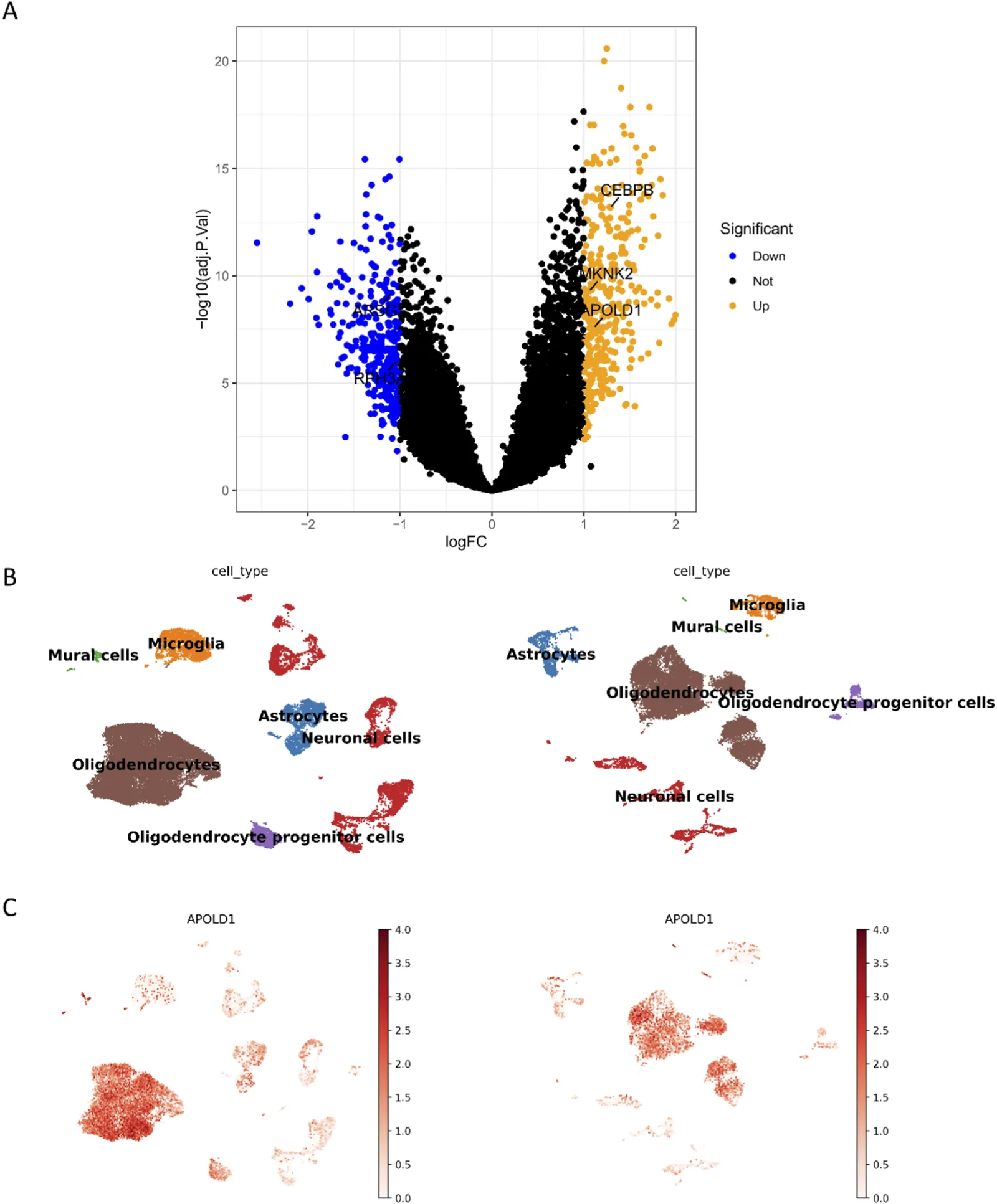
**(A)** The expression trends of APOLD1, RPH3A, ARSG, CEBPB, and MKNK2 in the bulk brain transcriptomics. **(B)** Left: Visualization of cell types in the AD group. Right: Visualization of cell types in the control group. **(C)** Left: Visualization of APOLD1 expression in the AD group. Right: Visualization of APOLD1 expression in the control group.

In this study, we utilized brain tissue transcriptomic data to obtain gene weights through the gene selection module, and visualized the intersecting genes across all cross-validation folds (detailed in Supplementary Figure S1). Based on their weight proportions, the top 23 high-weight genes selected by DeepFS were prioritized as candidate genes for subsequent biological validation. These selected genes were subsequently cross-referenced against published literature, with their detailed comprehensive information documented in Supplementary Table S1. Combining the aforementioned genes with the snRNA-seq data, a quantitative statistical evaluation was conducted by running cell-type-specific DEA between AD and control subjects across all cell types. This process successfully isolated genes with statistically significant variations, as documented in Table 5. It can be observed that 5 genes still exhibited cell-type-specific perturbations at single-cell resolution. To further elucidate the cross-modal biological relevance of the genes selected by DeepFS, we visualized the expression trends of APOLD1, RPH3A, ARSG, CEBPB, and MKNK2 in the bulk brain transcriptomics using a volcano plot (Figure 6A). The results demonstrated that APOLD1, CEBPB, and MKNK2 were significantly upregulated, whereas RPH3A and ARSG were significantly downregulated. Crucially, these expression trends at the bulk tissue level were highly consistent with the direction of cell-type-specific transcriptional dysregulation observed in the snRNA-seq dataset. Among them, APOLD1 showed varying degrees of significant difference across 4 cell types. We visualized the expression of APOLD1 as shown in Figure 6C. Concurrently, published literature has also demonstrated the relationship between the aforementioned genes and AD. For instance, Yao et al. demonstrated that by mutating cleavage sites to block δ-secretase cleavage of amyloid precursor protein, the truncated amyloid precursor protein fragment C586-695 produced by δ-secretase directly binds to CEBPB, exacerbating AD-related gene expression and pathogenesis. This highlights the critical role of CEBPB in the development of AD (Yao et al., 2021). Cai et al. constructed a competing endogenous RNA network and performed enrichment analysis, inferring that the two lncRNAs, LINC00365 and FBXL19-AS1, may regulate the expression of MKNK2 and influence the progression of AD (Cai et al., 2023). Doan et al. integrated multiple AD transcriptome data from 19 candidate genes and found that RPH3A is downregulated in the synaptic signaling pathway, which was demonstrated in a further mouse model (Nguyen et al., 2024). This independent validation experiment, combined with literature analysis, deeply confirms the biological validity of the gene selection module proposed in this study. Given the small number of genes (n = 5) that showed significant cell-type-specific differential expression in the independent snRNA-seq dataset, conventional pathway enrichment analysis was not performed due to limited statistical power.

**TABLE 5:** Cell-type-specific significantly differentially expressed genes.

Gene	Cell type	log2FC	P_adj
APOLD1	Oligodendrocytes	0.8754	1.8702e-117
APOLD1	Oligodendrocyte progenitor cells	1.4588	4.0325e-6
APOLD1	Neuronal cells	0.5875	0.0002
APOLD1	Astrocytes	0.7582	0.0009
RPH3A	Neuronal cells	−0.3816	3.0390e-12
ARSG	Neuronal cells	−0.3797	9.8343e-6
CEBPB	Microglia	0.6316	8.0284e-5
CEBPB	Astrocytes	0.7542	0.0077
MKNK2	Oligodendrocytes	0.5192	0.0002
MKNK2	Astrocytes	0.5737	0.0003

## 4 Conclusion

In this study, we designed DeepFS, a framework based on gene selection, to realize the diagnosis of AD based on transcriptomic data, firstly, we performed DEA for the gene expression matrix obtained by preprocessing, and used the differential expression matrix as the input of DeepFS for classification, and obtained an accuracy rate of 89.45% and an AUC value of 0.9647, which proved the DeepFS’s ability to classify transcriptome data. Subsequently, by conducting independent validations on the human peripheral blood transcriptomic dataset and the brain tissue snRNA-seq dataset, this study demonstrated the methodological generalizability of the framework across different tissue types and the biological validity of the gene selection module, respectively. The true advantage of DeepFS lies in its capacity to contract the candidate space, providing a prior reference for subsequent research on critical AD biomarkers.

The limitations of this study include the limited number of incorporated datasets, the lack of an independent external bulk transcriptomic cohort of brain tissue for validation, and the inadequate adjustment for important confounding factors (e.g., age, sex, and brain region). These factors may influence gene expression patterns independently of AD status and may therefore contribute to the observed transcriptional differences and model performance. For example, age-related transcriptional changes may overlap with molecular alterations associated with AD, while sex differences may influence gene expression and disease-related biological processes. In addition, substantial transcriptional heterogeneity exists across different brain regions, and region-specific expression patterns may introduce additional variation that is not directly related to AD status. Future studies should further collect or integrate brain-tissue transcriptomic data from different research centers and independent cohorts to validate the generalizability of DeepFS using entirely independent datasets, while also evaluating the effects of differences in sequencing platforms, experimental procedures, and population characteristics on model performance. This will help further establish the robustness and practical applicability of DeepFS across different brain-tissue transcriptomic datasets.

## Data Availability

The original contributions presented in the study are included in the article/Supplementary Material, further inquiries can be directed to the corresponding author.

## References

[B1] Amoroso N. Diacono D. Fanizzi A. La Rocca M. Monaco A. Lombardi A. (2018). Deep learning reveals Alzheimer's disease onset in MCI subjects: results from an international challenge. J. Neuroscience Methods 302, 3–9. 10.1016/j.jneumeth.2017.12.011 29287745

[B2] Anders S. Huber W. (2010). Differential expression analysis for sequence count data. Nat. Preced. 10.1038/npre.2010.4282.1 PMC3218662 20979621

[B3] Andrási E. Farkas É. Scheibler H. Réffy A. Bezúr L. (1995). Al, Zn, Cu, Mn and Fe levels in brain in Alzheimer's disease. Archives Gerontology Geriatrics 21(1), 89–97. 10.1016/0167-4943(95)00643-Y 15374228

[B4] Ausó E. Gómez-Vicente V. Esquiva G. (2020). Biomarkers for alzheimer’s disease early diagnosis. J. Personalized Med. 10 (3), 114. 10.3390/jpm10030114 32899797 PMC7563965

[B5] Cai H.-Y. Chen S.-R. Wang Y. Jiao J.-J. Qiao J. Hölscher C. (2023). Integrated analysis of the lncRNA-associated ceRNA network in Alzheimer's disease. Gene 876, 147484. 10.1016/j.gene.2023.147484 37187245

[B6] Cao Z. Du Y. Xu G. Zhu H. Ma Y. Wang Z. (2024). From diabetes to dementia: identifying key genes in the progression of cognitive impairment. Brain Sci. 14 (10), 1035. 10.3390/brainsci14101035 39452046 PMC11506463

[B7] Consortium T. G. O. Aleksander S. A. Balhoff J. Carbon S. Cherry J. M. Drabkin H. J. (2023). The gene ontology knowledgebase in 2023. Genetics 224 (1), iyad031. 10.1093/genetics/iyad031 36866529 PMC10158837

[B8] Huang Y. Xu J. Zhou Y. Tong T. Zhuang X. and t. A. s. D. N. I. (2019). Diagnosis of Alzheimer’s disease *via* multi-modality 3D convolutional neural network. Front. Neurosci. 13, 13–2019. 10.3389/fnins.2019.00509 31213967 PMC6555226

[B9] Hung Y. H. Bush A. I. Cherny R. A. (2010). Copper in the brain and Alzheimer’s disease. JBIC J. Biol. Inorg. Chem. 15 (1), 61–76. 10.1007/s00775-009-0600-y 19862561

[B10] Hureau C. Faller P. (2009). Aβ-mediated ROS production by Cu ions: structural insights, mechanisms and relevance to Alzheimer's disease. Biochimie 91(10), 1212–1217. 10.1016/j.biochi.2009.03.013 19332103

[B11] Iturria-Medina Y. Adewale Q. Khan A. F. Ducharme S. Rosa-Neto P. O’Donnell K. (2022). Unified epigenomic, transcriptomic, proteomic, and metabolomic taxonomy of Alzheimer’s disease progression and heterogeneity. Sci. Adv., 8(46), eabo6764. 10.1126/sciadv.abo6764 PMC9674284 36399579

[B12] Ji H. Liu Z. Yan W. Q. Klette R. (2019). Early Diagnosis of Alzheimer's disease Using Deep Learning Proceedings of the 2nd International Conference on Control and Computer Vision. Republic of Korea: Jeju, 87–91. 10.1145/3341016.3341024

[B13] Kanehisa M. Furumichi M. Tanabe M. Sato Y. Morishima K. (2017). KEGG: new perspectives on genomes, pathways, diseases and drugs. Nucleic Acids Res. 45 (D1), D353–D361. 10.1093/nar/gkw1092 27899662 PMC5210567

[B14] Kokkinou M. Beishon L. C. Smailagic N. Noel-Storr A. H. Hyde C. Ukoumunne O. (2021). Plasma and cerebrospinal fluid ABeta42 for the differential diagnosis of Alzheimer's disease dementia in participants diagnosed with any dementia subtype in a specialist care setting. Cochrane Database Syst. Rev. 2 (2). 10.1002/14651858.CD010945.pub2 33566374 PMC8078224

[B15] Liu T. Li Y. (2023). scCGImpute: an imputation method for single-cell RNA sequencing data based on similarities between cells and relationships among genes. Appl. Sci. 13 (13), 7936. 10.3390/app13137936

[B16] Nguyen D. P. Q. Pham S. Jallow A. W. Ho N.-T. Le B. Quang H. T. (2024). Multiple transcriptomic analyses explore potential synaptic biomarker Rabphilin-3A for Alzheimer's disease. Sci. Reports 14 (1), 18717. 10.1038/s41598-024-66693-8 39134564 PMC11319786

[B17] Porsteinsson A. P. Isaacson R. S. Knox S. Sabbagh M. N. Rubino I. (2021). Diagnosis of early Alzheimer’s disease: clinical practice in 2021. J. Prev. Alzheimer's Dis. 8 (3), 371–386. 10.14283/jpad.2021.23 34101796 PMC12280795

[B18] Ritchie M. E. Phipson B. Wu D. Hu Y. Law C. W. Shi W. (2015). limma powers differential expression analyses for RNA-sequencing and microarray studies. Nucleic Acids Res. 43 (7), e47. e47-e47. 10.1093/nar/gkv007 25605792 PMC4402510

[B19] Tatulian S. A. (2022). Challenges and hopes for Alzheimer’s disease. Drug Discov. Today 27 (4), 1027–1043. 10.1016/j.drudis.2022.01.016 35121174

[B20] Traag V. A. Waltman L. van Eck N. J. (2019). From Louvain to Leiden: guaranteeing well-connected communities. Sci. Reports 9 (1), 5233. 10.1038/s41598-019-41695-z 30914743 PMC6435756

[B21] Vaz M. Silvestre S. (2020). Alzheimer's disease: recent treatment strategies. Eur. J. Pharmacol. 887, 173554. 10.1016/j.ejphar.2020.173554 32941929

[B22] Vlachos G. S. Kosmidis M. H. Yannakoulia M. Dardiotis E. Hadjigeorgiou G. Sakka P. (2020). Prevalence of mild cognitive impairment in the elderly population in Greece: results from the HELIAD study. Alzheimer Dis. and Assoc. Disord. 34 (2), 156–162. 10.1097/WAD.0000000000000361 31913961

[B23] Wang M. Gan J. Han C. Guo Y. Chen K. Shi Y.-z. (2022a). Imputation methods for scRNA sequencing data. Appl. Sci. 12 (20), 10684. 10.3390/app122010684

[B24] Wang Q. Chen K. Su Y. Reiman E. M. Dudley J. T. Readhead B. (2022b). Deep learning-based brain transcriptomic signatures associated with the neuropathological and clinical severity of Alzheimer’s disease. Brain Commun. 4 (1), fcab293. 10.1093/braincomms/fcab293 34993477 PMC8728025

[B25] Wang H. Li J. Tu W. Wang Z. Zhang Y. Chang L. (2024). Identification of blood biomarkers related to energy metabolism and construction of diagnostic prediction model based on three independent Alzheimer’s disease cohorts. J. Alzheimer’s Dis. 100 (4), 1261–1287. 10.3233/jad-240301 39093073 PMC11380308

[B26] Yao Y. Kang S. S. Xia Y. Wang Z.-H. Liu X. Muller T. (2021). A delta-secretase-truncated APP fragment activates CEBPB, mediating Alzheimer’s disease pathologies. Brain 144 (6), 1833–1852. 10.1093/brain/awab062 33880508 PMC8320270

[B27] Zetterberg H. (2019). Blood-based biomarkers for Alzheimer’s disease—An update. J. Neuroscience Methods, 319, 2–6. 10.1016/j.jneumeth.2018.10.025 30352211

[B28] Zhang F. Li Z. Zhang B. Du H. Wang B. Zhang X. (2019). Multi-modal deep learning model for auxiliary diagnosis of Alzheimer’s disease. Neurocomputing, 361, 185–195. 10.1016/j.neucom.2019.04.093

[B29] Zhang J. Liu T. Wu H. Wei J. Qu Q. (2025). Identification of NDUFV2, NDUFS7, OPA1, and NDUFA1 as biomarkers for Alzheimer's disease: insights from oxidative stress and mitochondrial dysfunction in the hippocampus. J. Alzheimer’s Dis. 106 (1), 167–183. 10.1177/13872877251339771 40329774

[B30] Zhao X. Hu J. Sun G. Fu J. He J. Jin X. (2024). Single-cell RNA sequencing and multiple bioinformatics methods to identify the biomarkers of ischemic stroke to alzheimer’s disease. Biomed. Signal Process. Control, 96, 106601. 10.1016/j.bspc.2024.106601

