# Supplementary material for: Alzheimer’s disease diagnosis based on gene selection and cross-tissue validation
Source: Front Genet. 2026 Sep 28;17:1890236. doi: 10.3389/fgene.2026.1890236 (PMC13654589; doi:10.3389/fgene.2026.1890236)
Supplement: Supplementary file 1 [file DataSheet1.pdf]

Table s1. Genes with higher weight in the gene selection module

	Gene	Evidence (PMID)
**1**	ZNF721	unconfirmed
**2**	CTD-3092A11.2	unconfirmed
**3**	CEBPB	33880508
**4**	LOC102724884	unconfirmed
**5**	SDR16C5	unconfirmed
**6**	MKNK2	37187245
**7**	ARSG	unconfirmed
**8**	GALNT15	37188790
**9**	ID3	37668961
**10**	APOLD1	unconfirmed
**11**	SEPP1	25298198
**12**	RPH3A	39134564
**13**	MAEL	unconfirmed
**14**	ELMO1	unconfirmed
**15**	OR7E47P	unconfirmed
**16**	CRYBA2	23709342
**17**	AP000265.1	27913295
**18**	KRTAP10-11	unconfirmed
**19**	TRIM17	35233562
**20**	ITFG2	unconfirmed
**21**	PLA2G4A	38528586
**22**	RP11-521O16.2	unconfirmed
**23**	TYRP1	38916996

Table s2. Permutation test results

Fold	Acc	Sen	Spec	Prec	MCC	F1	AUC
1	45.45%	63.16%	21.43%	52.17%	-16.58%	57.14%	0.3647
2	56.25%	64.71%	46.67%	57.89%	11.56%	61.11%	0.6392
3	46.88%	69.23%	31.58%	40.91%	0.86%	51.43%	0.5142
4	50.00%	47.62%	54.55%	66.67%	2.06%	55.56%	0.5368
5	59.38%	58.82%	60.00%	62.50%	18.79%	60.61%	0.6431
Avg	51.59%	60.71%	42.85%	56.03%	3.34%	57.17%	0.5396

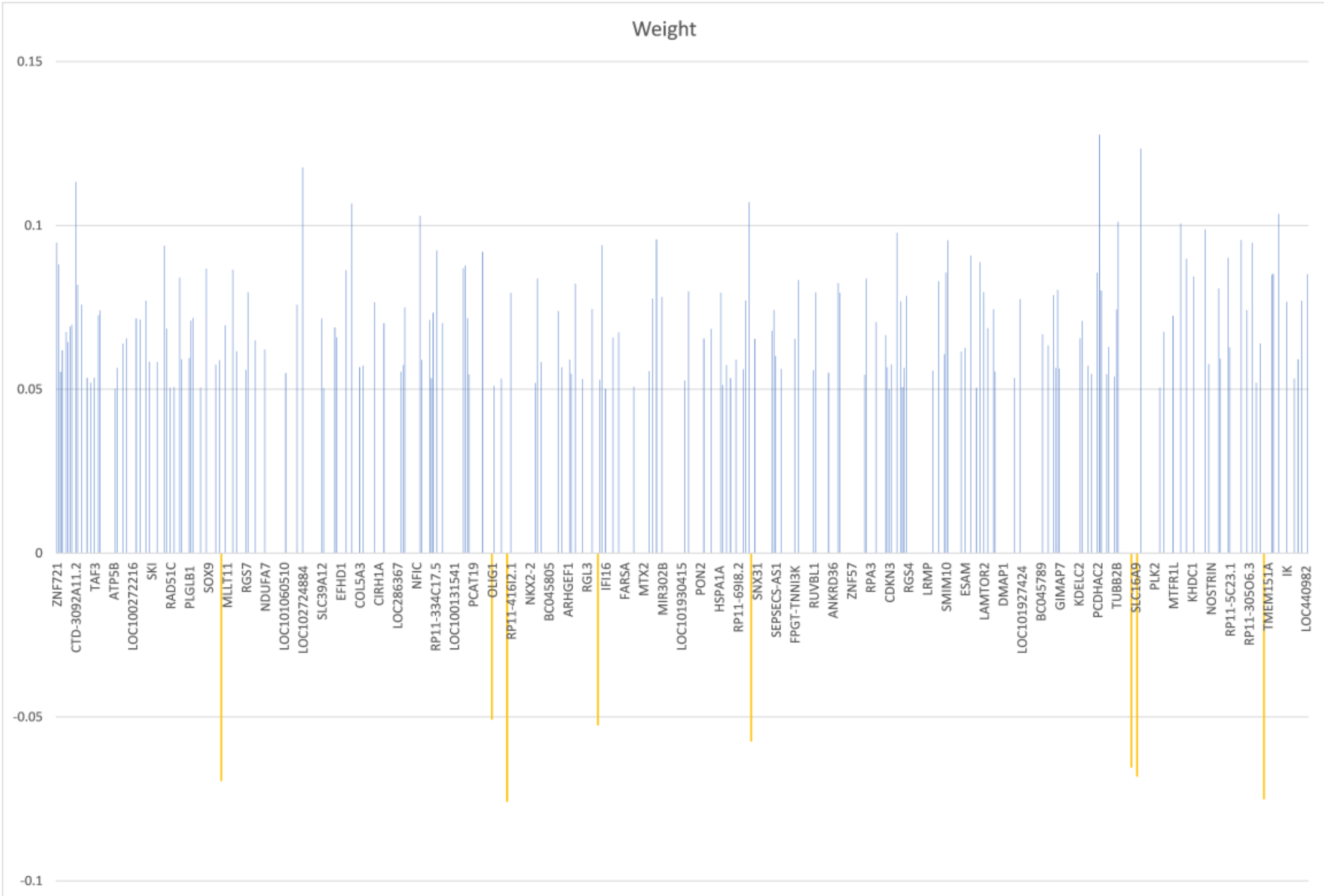

Fig s1. Gene weight bar chart
